# Analysis of Quasi-Simultaneous Laser Welding in T-Joint Configuration for PMMA-ABS Using Circular Wobble Geometry

**DOI:** 10.3390/ma18214819

**Published:** 2025-10-22

**Authors:** Antonio Lezzoche, Giulia Mossotti, Carmelo Nicosia, Marco Baggi, Michele Perlo, Luciano Scaltrito, Andrea Ancillao

**Affiliations:** 1 Dipartimento di Scienza Applicata e Tecnologia (DISAT), Politecnico di Torino, Corso Duca degli Abruzzi 24, 10129 Torino, Italy; 2 Dipartimento di Scienze e Innovazione Tecnologica (DISIT), Università degli Studi del Piemonte Orientale “Amedeo Avogadro”, Viale Teresa Michel 11, 15121 Alessandria, Italy; 3 CEMAS Elettra S.r.l., 10022 Carmagnola, Italym.perlo@cemaselettra.com (M.P.)

**Keywords:** laser welding, laser transmission welding, quasi simultaneous welding, ABS, PMMA, T-joint configuration, circular wobble, linear energy density, weld strength, penetration depth

## Abstract

The focus of this study was the investigation of the quasi-simultaneous laser welding (QSW) technique of polymethyl methacrylate (PMMA) and acrylonitrile butadiene styrene (ABS) in a T-joint configuration using a circular wobble laser path. The main aim was to find how laser parameters, such as scanning speed, number of scans, and laser power, influence key indicators of weld quality: penetration depth and weld strength. A range of scanning speeds (1–2 m/s) and scan repetitions (20–70) was explored, with the goal of keeping the total welding time around 1 s, a time compatible with industrial mass production. The results demonstrated a clear correlation between linear energy density and penetration depth. Deeper penetrations were achieved at higher energy levels. Weld strength was maximized with a lower number of scans (20) and higher powers (above 130 W). The configuration offering the best combination of weld strength (1137 N) and total welding time (0.8 s) was identified, demonstrating the suitability of QSW for mass production.

## 1. Introduction

Plastic welding has garnered increasing attention in recent years, driven by growing interest from a variety of industrial sectors, including automotive, biomedical, mechanical, and electronics. Among the various technologies employed for this purpose, laser transmission welding (LTW) has emerged as the most prominent, owing to its significant advantages over alternative methods, as highlighted in a recent review by Elsheikh, A. et al. [1]. These include the ability to minimize adverse thermal, mechanical, and electrical effects during the welding process. Within the domain of industrial mass production, one of the key quality indicators for a welding process is the welding time [2]. Thus, to optimize both production efficiency and cost, achieving high-quality results in shorter times is essential. In LTW, a laser beam is directed onto the top component, which typically has a lower absorption coefficient than the bottom component [3]. The light is then absorbed by the absorbent material and turned into heat. As the temperature rises, the absorbent material melts at the interface, forming a weld joint [4]. In 2012 Acherjee B. et al. [5] presented a comprehensive review about the LTW between dissimilar polymers, polymethyl methacrylate (PMMA) and acrylonitrile butadiene styrene (ABS), with a focus on the influence of process parameters. The configuration used for the welding was the overlap joint, which is the configuration used most often for LTW. It involves placing the two materials to be welded directly in contact, one stacked upon the other [6]. The authors highlighted the importance of parameters such as laser power, scanning speed, stand-off distance, and clamping on the weld morphology and weld strength., e.g., increasing laser power resulted in an increased joining area and a wider weld seam. Lower scan speeds led to excessive heat and surface degradation, while higher speeds led to insufficient heating and incomplete or weak weld. The optimal observed strength was 89 N/mm, measured by means of lap-shear test [5]. A similar study was conducted by Kucukoglu et al. in 2023 [7]. These authors also examined the effects of LTW between ABS and PMMA, aiming to optimize the manufacturing processes for automotive taillight components. For this research, a combination of Taguchi and TOPSIS methods was employed, using a diode laser welding machine (980 nm). The process parameters were optimized based on weld strength, breaking strain, and weld width. The best welding conditions were achieved with 50 W laser power, 100 mm/s welding speed, and a 150 N clamping force. The authors concluded that LTW proved to be a reliable method for the industrial welding of ABS and PMMA, offering advantages such as precision, repeatability, and suitability for high-volume automotive manufacturing [7].

Moreover, the topic of laser welding two transparent materials has also gained significant attention in recent times. This methodology is highly in demand by the biomedical sector and other sensitive industries, which often need to avoid the use of laser-absorbing materials [8]. In this configuration, both components to be joined are transparent to the laser beam, which complicates the generation of heat at their interface. To address this challenge, two main strategies are currently employed, as highlighted in the 2022 review by Haque et al. [6]:

(i) The use of Near-Infrared (NIR) Absorbing Additives as intermediate layers. In this approach, the additive placed at the joint interface absorbs the laser energy, generating the necessary heat to melt and weld the two transparent components. This method typically utilizes conventional laser frequencies (0.78 µm to 1.1 µm).
(ii) The use of lasers with tailored wavelengths (ranging from 1.4 µm to 2.2 µm). These longer wavelengths exploit the intrinsic molecular absorption bands of many clear polymers, allowing the required energy to be absorbed without the need for any external additives.

Among all the LTW techniques, the most used are (i) contour, (ii) simultaneous, and (iii) quasi-simultaneous welding. In contour laser welding (CW), the laser beam follows the predefined weld path a single time. This method typically requires low scanning speeds and high laser power, as the laser must locally heat and weld the materials as it moves along the joint line [9]. In simultaneous laser welding (SW), the entire weld geometry is fused at once. This requires a custom-designed optical system that distributes the laser energy across the full weld pattern. A 2020 study conducted by Catania et al. [10] investigated the effectiveness of SW in automotive applications. The authors developed a setup aimed at improving the speed and quality of laser plastic welding for large, complex parts. A hollow core waveguide was successfully integrated into the SW process to optimize laser energy distribution. By using a Taguchi design of experiment, they evaluated key parameters (exposure time, pressure, energy dose) and demonstrated the setup’s ability to achieve robust welds, offering a high-precision alternative to traditional joining methods for industrial production. While effective for high-throughput production, such a method lacked flexibility, as also reported in another subsequent study [11]. Quasi-simultaneous laser welding (QSW) represents a compromise between the previous two approaches. In this method, the laser rapidly scans the weld path multiple times in succession. This allows for repeated heating of the joint line, enabling uniform melting without the need for a complex and costly optical system. At the same time, it helps mitigate thermal stresses commonly associated with the contour method [12]. In 2003, Jansson A. et al. investigated the quality of QSW between polypropylene (PP) and polycarbonate (PC) by studying the correlation between the laser parameters (energy, scan speed, number of scans) and the measured dimension of the weld seam, while using an overlap configuration [13]. The main findings were that the thickness and width of the seam both increased with increased laser energy and increased number of scans. The tensile strength also increased with laser energy, and increasing the number of scans allowed them to further increase the strength per length. In the same study, it was observed that QSW allowed for a larger air gap between the materials compared to CW. A welding time of 1 s was identified as an acceptable threshold for industrial applications and was therefore adopted and recommended as a target duration for the experiments conducted [13]. In 2019, Acherjee [14] developed a 3D finite element model to simulate the heat transfer phenomena that occurs during QSW of thermoplastics. The studied material was Polycarbonate (PC), which they used in two different forms. A transparent PC plaque was used as the top material, and an absorbing PC plaque with the addition of 0.2% carbon black by weight was used as the bottom one. The study demonstrated that laser power, scanning speed, and the number of passes affected temperature distribution and weld seam formation. In the same paper, the author demonstrated that the weld zone temperature increased with the number of scans because the residual heat was not fully dissipated between rapid passes. This mechanism explained the interdependence of parameters: a low number of scans required significantly higher laser power to instantaneously reach the melting temperature, compensating for the minimal accumulated heat. Conversely, increasing the scan speed reduced the instantaneous heat input, necessitating a corresponding increase in power or in number of scans to maintain the required melt pool temperature and achieve a stable, high-quality weld [14]. In 2022, Jankus et al. [15] investigated the effects of meltdown on the quality of polymer joints produced by QSW. The authors correlated the reduction in the material due to the melting with the joint strength of the weld. Thermoplastic samples with three different laser transparencies were welded. Burst pressure tests were performed to evaluate the weld strength of the samples. The researchers discovered that meltdown was a critical and quantifiable factor that directly affected the weld. In fact, as the meltdown increased, the volume of molten polymer at the interface increased too. The study reported an optimal meltdown value (around 0.30 mm), after which excessive heat input could cause material degradation and a subsequent reduction in joint strength. In another study, by De Maddis et al. in 2025 [16], the integration of a robotic arm to replace the galvanometric heads typically used in quasi-simultaneous welding (QSLW) was explored. The authors investigated how key factors such as (i) laser power, (ii) clamping force, (iii) welding speed, and (iv) laser spot size could impact the meltdown phenomenon. The laser power required careful calibration: if too low, it could lead to incomplete material fusion; if too high, it could cause damage and material degradation. Similarly, a clamping force that is not sufficiently high to keep the materials in contact will prevent proper joining. The total meltdown was also shown to be dependent on the number of scans, increasing as the scan count rises. Finally, the laser spot size was directly related to energy density or fluence. For a constant power, a smaller laser spot concentrated the energy into a reduced area, leading to faster and more localized material heating. Conversely, a larger laser spot distributed the energy over a wider surface, thus requiring higher power levels to bring the affected material to its melting temperature. The best results in this study were associated with a velocity of 550 mm/s, a clamping force in the range of (60–80) N, and a spot size of 1.9 mm. Moreover, the use of a robotic arm was useful to control the main process parameters. It allowed the researchers to achieve high precision, repeatability, and flexibility.

In the industrial field, the use of the welding configuration known as a T-joint is increasingly gaining traction. This configuration consists of the orthogonal penetration of the protruding flange of the transparent material into the surface of the absorbing material. The advantages demonstrated by this configuration include improved stress distribution and easier characterization of the joint quality [17]. In the 2002 study conducted by Potente et al. [18], samples made of Polyether ether ketone (PEEK) and Polycarbonate (PC) were welded using quasi-simultaneous welding (QSW) in a T-joint configuration. The correlation between flange penetration depth into the absorbing part and weld strength was investigated, revealing that the best results were obtained with a penetration depth of 1 mm. The same penetration value was later confirmed for a different material pair, namely PMMA and ABS [19]. Most of the recent literature related to PMMA-ABS welding focuses on the overlapping joint method, while a detailed characterization of the T-joint in LTW and, more specifically, QSW, is required. A close study is that of Ilie M. et al. [20], focused on the weldability of ABS-ABS using LTW in a T-joint configuration. The samples were welded by means of a diode laser (940 nm) and the weld joint was tested for tensile strength. The maximum load obtained (2911 N) was achieved with a power of 15 W, a scanning speed of 0.01 m/s, a clamping pressure of 0.4 MPa, and two passes. Given the low number of passes, it is difficult to compare these results to the modern QSW, where the number of passes is significantly higher and the most common working speeds are in the order of 1 m/s. Further studies on the QSW are therefore necessary.

In modern LTW, a new approach known as the “Laser wobble welding process” has been gaining increasing recognition due to its distinctive features and advantages over the standard linear configuration. These include a wider weld seam, improved tolerance to air gaps, and a reduced occurrence of cracks [21,22]. A comprehensive review of wobble processes was proposed by Sanati et al. [23] in 2024. The most common wobble modes are circular, infinite, eight-shaped, linear, and sinusoidal modes. The circular mode exhibited the largest tensile strength, and this was attributed to the reduced likelihood of cracking.

The circular wobble configuration was also studied by Das et al. [24], who welded two sheets of aluminum alloy AW1050A with different thicknesses using a fillet edge weld.

## 2. Study Aim

To the best of our knowledge, no previous studies have investigated in depth the QSW with wobble passes of ABS and PMMA in a T-joint configuration with reference to the weld strength, laser parameters, and their optimization for massive industrial applications. Thus, the aims of this study were as follows:

(1) To investigate the QSW process of PMMA and ABS by verifying the relationship between penetration depth and the chosen set of laser process parameters;
(2) To identify the optimal welding parameters for such materials to provide recommendations for using this technique at an industrial level.

For such a study, (i) a T-joint welding configuration was adopted; (ii) the weld penetration depth was studied in relation to the welding time and energy input; and (iii) the welding forces of samples were compared under the same penetration depth and at different welding parameters.

## 3. Materials and Methods

### 3.1. Samples

For this study we tested PMMA samples (PLEXIGLAS^®^ 8N) (https://www.roehm.com, accessed on 7 October 2025) as the upper/transparent component, welded against ABS samples (Novodur HH-112 https://www.ineos-styrolution.com/index.html, accessed on 7 October 2025) as the lower/absorbent component. The materials’ properties provided by the manufacturers are listed in Table 1 and Table 2.

**Figure 1:**
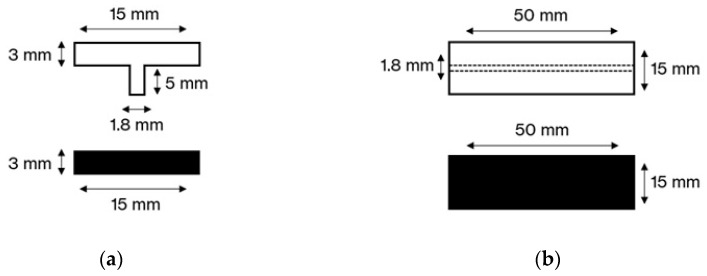
Schematic diagram of the samples: (**a**) front section of the material pair; (**b**) top view of the material pair.

The samples were shaped, respectively, as a T-bar and a flat bar, as illustrated in Figure 1. The T-joint was obtained at the bottom part of the T-bar.

### 3.2. Equipment

The tests were carried out using a custom-made QSW system based on a pig-tail fiber-coupled diode laser (BOX Optronics Tech, BLD-F975, Shenzhen, China, www.boxoptronics.com, accessed on 7 October 2025) with a maximum output power of 200 W. The diode laser operated at a wavelength of 975 nm, and a focal length of 650 mm was employed, resulting in a focal spot diameter of 1.7 mm over a working area of 70 mm × 70 mm. The laser beam was steered by means of a “Raylase Miniscan III-20” galvo scanner (Raylase, Wessling, Germany, www.raylase.de, accessed on 7 October 2025) and the system was controlled by the “Rayguide Click & Tech” software (Raylase, Wessling, Germany, www.raylase.de, accessed on 7 October 2025), which allowed us to draw and control the welding profile and parameters.

**Figure 2:**
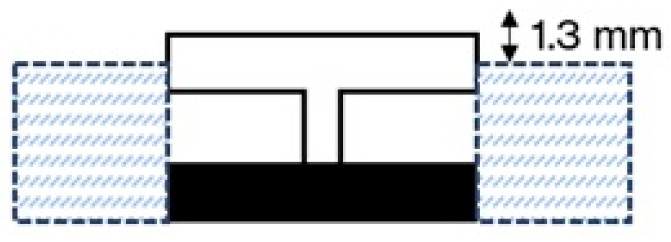
Schematic of the welding setup, including supports to keep the PMMA piece stable and fixed.

To ensure the stability of the material pair during the welding process, the transparent component, positioned above the absorbing component, was held in place using mechanical fixtures, as shown in Figure 2.

**Figure 3:**
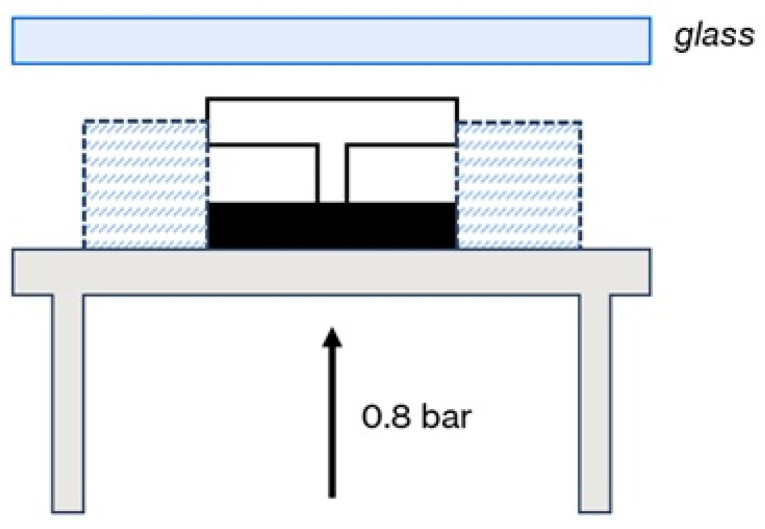
Summary setup.

The entire assembly rested on a metal plate connected to mechanical pistons, which, when activated, pressed the components against a glass plate positioned between the sample and the laser source. The applied pressure was set to 0.8 bar and maintained constant across the trials (Figure 3).

The penetration depth of the PMMA rib into the ABS block was measured using a pair of linear displacement transducers (CEMAS Elettra S.r.l., Carmagnola, Italy, cemaselettra.com, accessed on 7 October 2025) appropriately mounted on the sample holder support (accuracy ± 0.01 mm). Once the metal plate holding the samples was raised by the mechanical pistons and pressed against the glass surface, the zero-reference position was recorded. During the laser welding process, the transducers continuously monitored the vertical displacement of the ABS block. At the end of the process, the recorded displacement corresponded to the penetration depth of the PMMA into the ABS substrate.

**Figure 4:**
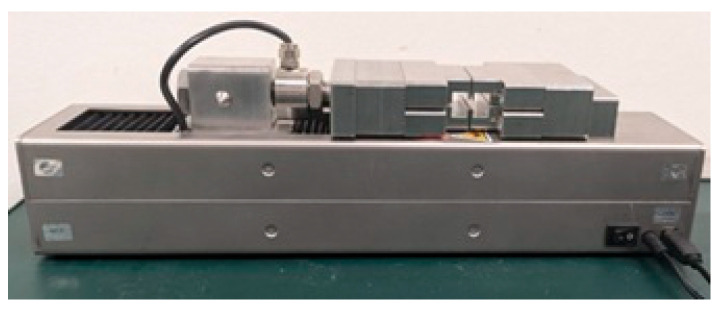
Mechanical testing device.

To assess the mechanical strength of the joints obtained via QSW, a custom-made tensile testing apparatus (CEMAS Elettra S.r.l., Carmagnola, Italy, cemaselettra.com, accessed on 7 October 2025) was employed. The device was specifically designed to accommodate samples in a T-joint configuration (Figure 4).

The welded sample was inserted into the fixture such that the absorbing block remained fixed, while the transparent rib was clamped and pulled vertically upwards. The machine applied a tensile load until joint failure occurred. The displacement rate of the system could be adjusted through a dedicated external software interface, allowing for control over the speed at which the load was applied. During each test, the applied force and the corresponding displacement were recorded continuously and exported as a CSV file. From these data, the maximum tensile force at failure (corresponding to the weld strength) was extracted for each sample.

### 3.3. Study Protocol

**Figure 5:**
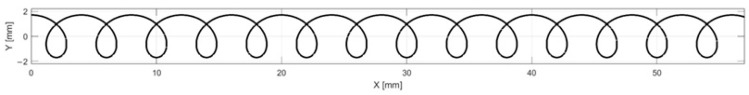
Schematic of a circular laser wobble trajectory with f = 1 kHz and a = 1.7 mm.

The testing campaign focused on investigating the penetration depth and bonding quality of the PMMA rib into the ABS block, with particular attention given to the welding cycle times. The outcomes were then correlated with the laser parameters set for each test. The measurement of the penetration depth was limited to the range (0.6–1.3) mm. The upper limit of 1.3 mm is due to the experimental setup (Figure 2). Since the width of the transparent rib section (1.8 mm) exceeds the laser spot diameter (1.7 mm), a circular wobble welding configuration was adopted to ensure full coverage of the joining surface. In this configuration, the laser beam does not follow a simple linear path but instead traces a helical trajectory as it moves along the joint. This was also recommended by previous studies to extend the diameter of the seam and improve the weld quality [17,18,19]. The wobble frequency and wobble amplitude were set to 1 kHz and 1.7 mm, respectively, obtaining a path as illustrated in Figure 5. The amplitude was chosen to be sure that the laser beam would fully cover the targeted surface area. The frequency of 1 kHz was selected because, as the frequency increases approaching this value, energy efficiency improves, while the heat affected zone (HAZ) and potential negative effects due to distortions are minimized, in line with findings reported in the literature [25,26]. In addition, the weld path length was set to 57 mm to ensure full coverage of the sample length.

Compared to a standard linear weld, a wobble weld acts over a greater total path length despite the same nominal weld length, due to the helical movement of the beam superimposed on its linear motion. This required the definition of a time correction factor, defined as the ratio between the wobble and linear welding times, to take into account the longer length. From experimental tests, the correction factor was found to consistently fall within the range of 1.2 to 1.4, indicating relatively stable behavior across all tested speed–repetition pairs.

Three different scanning speeds were evaluated (1 m/s, 1.5 m/s, and 2 m/s) along with four different repetition counts (20, 40, 50, and 70). The lower and upper limits (20 and 70 scans, respectively) were chosen according to the setup’s constraints to fully cover the required penetration depth range. The analysis was then extended to two intermediate values (40 and 50). For each set of parameters, three specimens were processed. The study consisted of three parts:

1. As a first step, the speed was kept constant, and the energy was increased linearly to define the interval producing a penetration depth in the defined range. This allowed us to check the variations in penetration depth with respect to the energy.
2. As a second step, the effect of the number of scans on penetration depth, at a fixed velocity, was investigated.
3. As a third step, the effect of scan velocity on penetration depth, at a fixed scan count, was investigated.

In all cases, the welding time was monitored in order to explore the possibility of reducing process time while maintaining consistent welding penetration performance.

**Figure 6:**
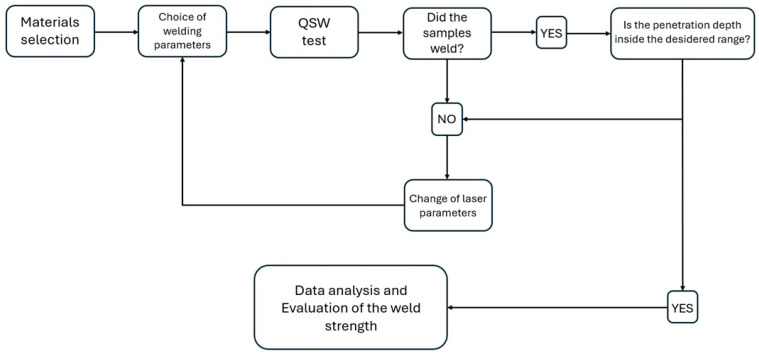
Overview diagram of the study workflow.

To test the welding forces, only the samples with a penetration depth of ~1 mm were included in the study. These were welded at the highest scanning speed (2 m/s), allowing for an analysis across four different scan number configurations (20, 40, 50, and 70). The penetration depth of T-joint configuration samples was recommended as a quality criterion and a penetration of ~1 mm was assumed as the threshold for successful welds [18]. The workflow is illustrated in Figure 6.

### 3.4. Data Analysis

The key laser process parameters that played a critical role in influencing weld performance in our study were as follows:

- The laser power output from the galvo head;
- The scanning speed of the laser beam;
- The number of repeated scans along the weld path.

Based on the previously mentioned parameters, the linear energy density of the welding process was calculated according to the formula proposed by Ghasemi et al. [12]:

(1) LET=E [J]L [m]=P W·ts[s]L [m]·Ns=P W·L mvs msL m·Ns=P [W]vs [ms]·Ns

The results that led to a penetration depth compatible with the range (0.6–1.3) mm were taken into consideration. The measured penetration depth values were averaged among the same samples, and their standard deviation was calculated. These values were then plotted against *LE_T_* and power (considering all combinations of speed and number of scans). Focusing on the samples that showed a penetration depth of around 1 mm, we measured the corresponding weld strengths. We averaged the results among the same samples and calculated their respective standard deviations. These values were then tabulated.

## 4. Results

### 4.1. Linear Energy Density (At Constant Velocity)

**Figure 7:**
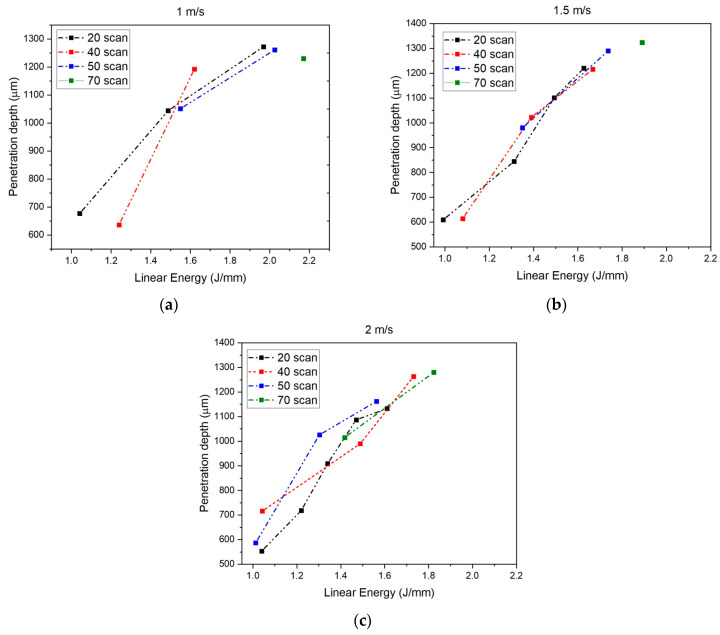
Linear energy related to penetration depth at different velocities: (**a**) 1 m/s; (**b**) 1.5 m/s; (**c**) 2 m/s.

The results of the study #1 are reported in Figure 7.

The penetration depth increased linearly with increasing linear energy delivered during the welding process. Particularly interesting were the trends observed in the processes with 70 scans. In these cases, the range of energy values reduced to a point for scanning speeds of 1 m/s and 1.5 m/s (Figure 7a,b). So, for these velocities, there is only one linear energy value that leads to a successful weld. In comparison, at 2 m/s, the range of linear energy values associated with 70 scans increased (Figure 7c).

For each combination of scans and velocity, the total welding time was indicated in Table 3. It was observed that it was possible to obtain the required penetration depth in less than 1 s, by using 2 m/s as the scanning speed and 20 scanning repetitions. This configuration is the most suitable for industrial mass production.

### 4.2. Power (At Constant Velocity)

**Figure 8:**
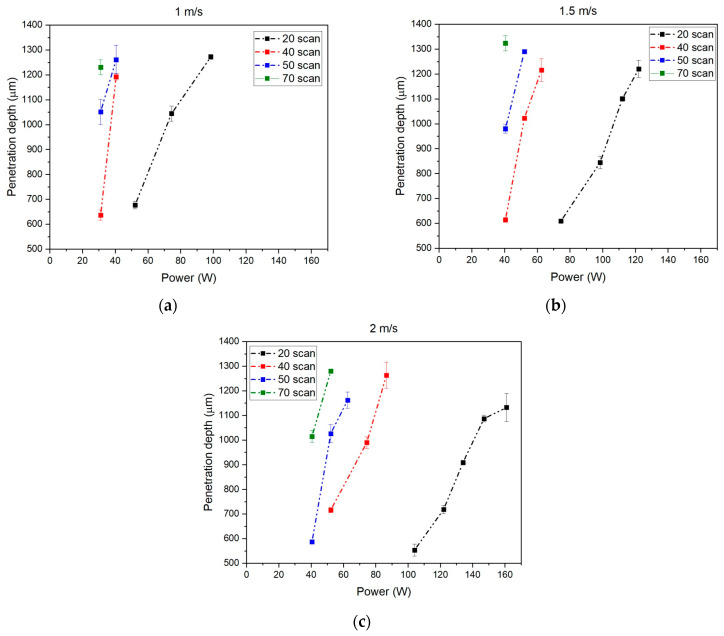
Effect of the power for different numbers of scans at different velocities: (**a**) 1 m/s; (**b**) 1.5 m/s; (**c**) 2 m/s.

Figure 8 shows the results of study #2.

At equal power levels, greater penetration depth was observed as the number of scans increased. A higher number of scans, at constant scanning speed, corresponded to a greater linear energy input, and consequently, to a greater weld penetration. As the scanning speed increased, it was observed that similarly to what was noted for linear energy, the curves associated with each number of scans spanned a wider range of power values (Figure 8). With a fixed velocity, it is possible to reach similar penetration depths with different number of scans. This is possible thanks to a suitable choice of power supply. It is important to point out that as the number of scans increases, the power range for a correct welding becomes smaller. This reduces the flexibility of the choice.

At the lowest scanning speed (1 m/s), it was observed that the power required to achieve the minimum penetration depth was the same for the processes with 40, 50, and 70 scans (Figure 8a). Additionally, the power range for 40 and 50 scans appears identical at 1 m/s (Figure 8a) and remains similar at 1.5 m/s (Figure 8b). In contrast, significant differences are observed for the processes with 20 scans, which require higher power levels to achieve the desired welds and penetration depths (Figure 8). At 2 m/s, the differences in required power become more pronounced across the scan number sets (Figure 8c).

### 4.3. Power (At Constant Number of Scans)

**Figure 9:**
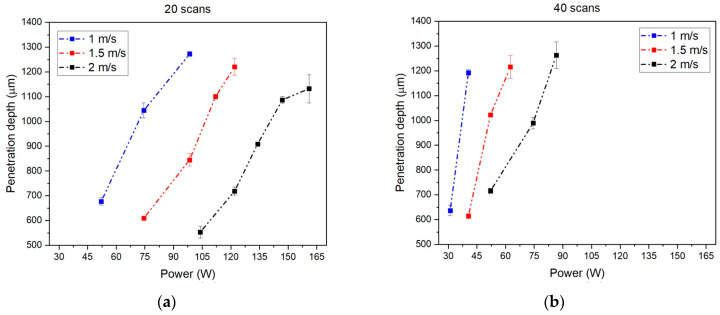

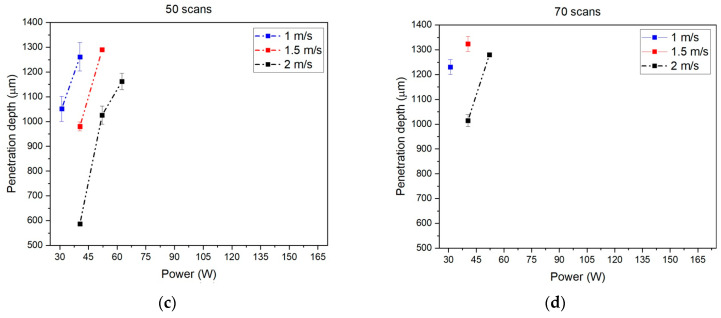
Effect of the power for different velocities at different numbers of scans: (**a**) 20; (**b**) 40; (**c**) 50; (**d**) 70.

Figure 9 shows the results of study #3.

Wider power ranges are observed when a lower number of scans is used (Figure 9a). As the number of scans increases, the number of power values that enable correct welding decreases. At the same power values, a decrease in scanning speed results in greater weld penetration. This occurred because, when the number of scans was kept constant, a slower laser movement produced a longer exposure and greater linear energy, leading to deeper penetration (Figure 9). This test showed that increasing the number of scans had a similar effect to decreasing the velocity, which is coherent with Equation (1). These curves showed that similar penetration depths could be achieved at different velocities by choosing an appropriate power. Higher speeds required higher power, still following Equation (1). The choice of powers reduced as the number of scans increased.

### 4.4. Weld Strength

Strength testing results are shown in Table 4.

The samples welded with 40, 50, and 70 scans exhibited comparable welding forces, all exceeding 900 N. The sample welded with 40 scans required significantly higher power input than the other two to achieve the desired penetration threshold, but it reached the same result while reducing the welding time. This effect became even more evident in the two samples welded with 20 scans, where the lower number of scans allowed us to achieve a penetration depth of about 1 mm using two different power values (and thus different linear energies). Both samples required very high powers to enable the process (134 W and 147 W, respectively) but yielded the highest absolute welding force values. Additionally, the welding time was only 0.8 s, well below that of all other tests. These cases were identified as the optimal parameter set for achieving the strongest weld.

## 5. Discussion

Our results demonstrated how laser process parameters influenced the weld quality. We first analyzed the effect of the linear energy density transferred (LE_T_). It was observed that increasing LE_T_ directly resulted in an increase in the penetration depth, which is in agreement with established literature [16]. We also found that similar penetration depths could be achieved using different parametric sets that yielded the same LE_T_. This suggested a significant degree of process flexibility, confirming that equivalent welding outcomes can be obtained through various combinations of laser power, scanning speed, and number of scans. This flexibility allowed for the collection of varying welding times, with the minimum recorded time reaching 0.8 s. Achieving these lower weld times (equivalent to higher speeds and scan numbers) necessarily required a corresponding increase in laser power input. The results demonstrated that for the highest number of scans (70) at lower welding speeds (1.0 and 1.5 m/s), the breadth of available options narrowed to a singular successful linear energy density. This was attributed to the thermal accumulation effect. The found value represented the upper boundary condition where the maximum permissible heat was injected, associated with the minimum required combination of laser power and speed. Further increasing the scan count led to material degradation.

Regarding laser power, we observed that an increase in power, while keeping speed and scan count constant, resulted in a marked increase in the penetration depth. This finding is consistent with the previous literature [3,16,26,27]. As reported by Ghasemi et al. [12], increasing power enhances the total linear energy transferred to the materials, leading to greater penetration. Higher velocities lead to a reduction in laser interaction time, thus requiring higher power levels to reach the melting temperature necessary for welding. Conversely, when the number of scans increased, both the total welding time and the exposure to the laser beam increased, resulting in a narrowing of the range of applicable power values to achieve melting and similar penetration depths. The same finding was also reported in another study [14].

Most of the previous ABS-PMMA welding studies focused on the overlap configuration, e.g., the study of Kucukoglu et al. [7] demonstrated the relevance and effectiveness of welding these materials, particularly in the automotive industry, due to the breaking force values obtained in their tests. The authors performed shear strength tests, reporting a maximum failure load of 1703.9 N for the welded joint. With regard to the welded area (186 mm^2^), this corresponded to a tensile strength of 9.16 N/mm^2^. Considering the relatively low welding speed reported (100 mm/s), such a procedure can be compared to a contour laser welding. In our study, we adopted a QSW and multi-pass approach that allowed us to reach a higher tensile strength (12.58 N/mm^2^), corresponding to a maximum joint failure force of 1132 N over a welded area of 90 mm^2^. The QSW method was investigated by Jannsen et al. [13] in relation to different material pairs (PP-PP and PC-PC) and with the overlap configuration. In this study, the observed tensile strength values were 23 N/mm^2^ for PC-PC and 33 N/mm^2^ for PP-PP. In this case, the results were closer to those observed in our tests.

The research conducted in 2009 by Ilie M. et al. [20] on T-joint ABS–ABS welding reported a maximum load of 2111 N. The study highlighted the importance of two factors in achieving the desired welding strength: laser power and clamping force. The authors reported the need to operate with low power (15 W) and high pressure (0.4 MPa) to maximize this result. The difference compared to our findings can be attributed to the different technologies employed. In fact, unlike our QSW process, their approach is closer to the contour technique. In further detail, the welding was carried out at a scanning speed (0.01 m/s) significantly lower than ours (2 m/s) and with two passes instead of the 20 or more used in our study. The total linear energy of the welding process reported in [16] amounted to 3 J/mm, i.e., approximately twice the value that we recorded in our samples that exhibited the maximum breaking strength.

A limitation of our study is that the influence of overheating and the formation of internal voids were not investigated. Further studies should include an in-depth analysis of the failure modes and of heat absorption in the materials, supported by computer simulations.

## 6. Conclusions

In this study, we investigated the QSW of ABS–PMMA sample pairs in a T-joint configuration. Penetration depth was found to be related to the linear energy input, which, in turn, depends on (i) laser power, (ii) scanning speed, and (iii) number of scans. We observed how each of these influenced the weld quality. A proper weld, with a penetration depth of about 1 mm, was obtained at the highest scanning speed (2 m/s) using different combinations of scans and laser power. Weld strength tests were carried out on the samples that reached a penetration depth of at least 1 mm. The best results (above 980 N) were associated with lower scan counts. The observed tensile strength was comparable to values reported in previous studies about LTW plastics materials also with different joint configurations, confirming the suitability of the QSW technique for this process. A welding time of 0.8 s proved to be well aligned with industrial requirements for mass production, indicating that QSW of plastics in a T-joint configuration could represent a valid alternative to other competitive laser welding techniques.

## Figures and Tables

**Table 1:** Main ABS properties (https://www.ineos-styrolution.com/index.html, accessed on 7 October 2025).

Melt Volume Rate 220 °C/10 kg	5.5 cm^3^/10 min
Tensile modulus	2700 MPa
Tensile stress at break, 23 °C	40 MPa
Coefficient of linear thermal expansion	9 × 10^−5^/°C
Thermal conductivity	0.17 W/(m·K)
Density	1050 kg/m^3^

**Table 2:** Main PMMA properties (https://www.roehm.com, accessed on 7 October 2025).

Melt Volume Rate	3 cm^3^/10 min
Tensile modulus	3300 MPa
Stress at break	77 MPa
Coefficient of linear thermal expansion	8 × 10^−6^/°C
Thermal conductivity	0.181 W/(m·K)
Density	1060 kg/m^3^

**Table 3:** Welding times associated with each scans–velocity pair.

Number of Scans	Velocity (m/s)	Range of Linear Energy (J/mm)	Welding Time (s)
20	1	(1.04–1.97)	1.35
40	1	(1.24–1.62)	2.7
50	1	(1.55–2.03)	3.4
70	1	2.17	4.8
20	1.5	(0.99–1.63)	0.98
40	1.5	(1.08–1.67)	1.98
50	1.5	(1.35–1.74)	2.47
70	1.5	1.89	3.46
20	2	(1.04–1.61)	0.8
40	2	(1.04–1.73)	1.59
50	2	(1.01–1.56)	1.99
70	2	(1.42–1.82)	2.8

**Table 4:** Weld Strength comparison at different power and scans with same penetration depth.

Velocity (m/s)	Scans	Power (W)	Linear Energy (J/mm)	Penetration Depth (mm)	Weld Strength (N)	Welding Time (s)
2	20	134	1.34	0.909 ± 0.002	1132 ± 12	0.8
2	20	147	1.47	1.087 ± 0.013	985 ± 15	0.8
2	40	74.4	1.49	0.989 ± 0.024	926 ± 16	1.59
2	50	52.1	1.30	1.026 ± 0.037	977 ± 17	1.99
2	70	40.5	1.42	1.015 ± 0.024	943 ± 18	2.8

## Data Availability

The original contributions presented in this study are included in the article. Further inquiries can be directed to the corresponding author.

## Abbreviations

The following abbreviations are used in this manuscript:

ABS	Acrylonitrile butadiene styrene
CW	Contour Laser welding
HAZ	Heat affected zone
LE_T_	Linear Energy transferred
LTW	Laser transmission welding
PC	Polycarbonate
PEEK	Polyether ether ketone
PMMA	Polymethyl methacrylate
PP	Polypropylene
QSW	Quasi-simultaneous laser welding
SW	Simultaneous laser welding
